# Protective Effect of Bergenin against Cyclophosphamide-Induced Immunosuppression by Immunomodulatory Effect and Antioxidation in Balb/c Mice

**DOI:** 10.3390/molecules23102668

**Published:** 2018-10-17

**Authors:** Qiuchen Qi, Zhonghua Dong, Yueyue Sun, Siying Li, Zhongxi Zhao

**Affiliations:** 1 School of Pharmaceutical Sciences, Shandong University, 44 West Wenhua Road, Jinan 250012, Shandong, China; qiuchensd@163.com (Q.Q.); 201514330@mail.sdu.edu.cn (Z.D.); 13021717075@163.com (Y.S.); 2 Department of Pathology and Pathophysiology, School of Basic Medical Sciences, Shandong University, 44 West Wenhua Road, Jinan 250012, China; li-siying@hotmail.com; 3 Shandong Engineering & Technology Research Center for Jujube Food and Drug, 44 West Wenhua Road, Jinan 250012, Shandong, China; 4 Shandong Provincial Key Laboratory of Mucosal and Transdermal Drug Delivery Technologies, Shandong Academy of Pharmaceutical Sciences, 989 Xinluo Street, Jinan 250101, Shandong, China

**Keywords:** bergenin, cyclophosphamide, cytokines, lymphocytes, immunomodulatory, antioxidant activity

## Abstract

In this study, the aim was to investigate the effect of bergenin on immune function and antioxidation in cyclophosphamide (Cy)-induced immunosuppressed mice. Firstly, we estimated its effect on immune organs. Histological analysis and indexes of immune organs showed that cyclophosphamide exhibited spleen and thymus injury compared with the normal control, which was alleviated by bergenin. Secondly, bergenin also enhanced the humoral immune function through increasing the level of IgM and IgG in serum. Thirdly, bergenin also enhanced the cellular immune function. The results indicate that bergenin increased peritoneal macrophage functions, the proliferation of T and B lymphocytes, NK and CTL cell activities, and T (CD4^+^ and CD8^+^) lymphocyte subsets. Besides, bergenin also had the ability to modulate the Th1/Th2 balance. Moreover, bergenin prevented the Cy-induced decrease in numbers of peripheral RBC, WBC and platelets, providing supportive evidence for their anti-leukopenia activities. Finally, bergenin also reversed the Cy-induced decrease in the total antioxidant capacity including activities of superoxide dismutase (SOD), catalase (CAT) and glutathione peroxidase (GSH-Px). In conclusion, bergenin protected against Cy-induced adverse reactions by enhancing humoral and cellular immune functions and augmenting antioxidative activity and could be considered as a potential immunomodulatory agent.

## 1. Introduction

The immune system plays an important role in defending against microbes and foreign antigens [1]. It is involved in the recovery from the diseases determined by the host’s immune status like cancer, obesity, diabetes, tuberculosis, etc. [2]. Thus, the body’s immune function is vitally important for prevention and recovery from these immune-mediated diseases. Therefore, to study and develop new immunomodulatory agents is one of the most effective methods for prevention and treatment of the diseases associated with immunosuppression. 

According to the WHO, cancer is the major leading cause of death around the world [3]. Generally, chemotherapeutics drugs often generate many harmful side effects such as cytotoxic effects and host immunosuppression, no matter how great of their therapeutic effects are [4]. Cyclophosphamide (Cy), an alkylating agent, has been widely used as chemotherapeutic drug which results in a lot of adverse effects including immunosuppression, bone marrow suppression, leucopenia and oxidative stress [5,6,7,8]. It has been reported that Cy can generate oxidative stresses, mediate disruption of the redox balance, and lead to biochemical and physiological disturbances [9]. Administration of Cy can damage the Th1/Th2 balance [10] and induce a reduction in the absolute counts of T cells and B cells [11]. Therefore, we established a murine model of immunosuppression in Balb/c mice with cyclophosphamide to investigate the immunomodulatory effect of bergenin.

In recent years, a variety of medicinal plants have been shown to possess modulating functions of the innate and adaptive immune systems and have anti-oxidative characteristics [12,13]. In fact, Chinese herbal medicines have been widely used to protect against the side-effects associated with chemotherapeutics drugs in cancer treatment [14,15]. Bergenin is an active constituent of the plants of the genus *Bergenia*, which have been traditionally used for treatment of diarrhea, vomiting, fever, cough, pulmonary infections and menorrhagia [16]. Bergenin shows a wide range of pharmacological activities such as anti-arrhythmic, anti-inflammatory, anti-malarial, neuro- protective, and anti-tumor properties, and is widely used in Traditional Chinese Medicines [17,18,19,20,21,22]. Bergenin either acts as a free radical scavenger or a redox regulatory agent. In addition, bergenin has anti-inflammatory activity through selectively inhibiting COX-2 and modulating of Th1/Th2 cytokine balance [23,24,25].

Despite all these reports on the effects of bergenin, its immunomodulatory activity roles in vivo were not investigated. Thus, this study was designed to assess the effect of bergenin on humoral and cellular immunity as well as antioxidation in immunosuppressed mice induced by Cy. The effects of bergenin were evaluated from immune organs, immune cell activity, serum cytokines, immunoglobulin (Ig) levels, peripheral RBC, WBC and platelet numbers as well as antioxidant activity in mice. 

## 2. Results

### 2.1. Histological Observations of Spleen and Thymus 

**Figure 1:**
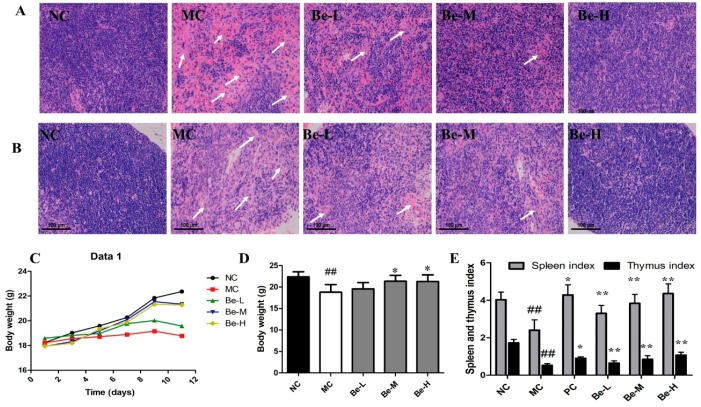
Effects of bergenin on spleen and thymus tissues in Cy-treated mice. (**A**) Spleen tissues in the HE stained histopathological images (objective: 100×). (**B**) Thymus tissues in the HE stained histopathological images (objective: 100×). (**C**) Body weights during treatment. (**D**) Body weights after treatment. (**E**) Immune organ indices. NC: normal control group, dosed with saline; MC: model control group, treated with cyclophosphamide; Be-L: challenged with Cy and treated with 5 mg/kg/day bergenin; Be-M: challenged with Cy and treated with 10 mg/kg/day bergenin; Be-H: challenged with Cy and treated with 20 mg/kg/day bergenin. Data are expressed as mean ± SD (*n* = 8). ^##^ *p* < 0.01 vs. the NC group, * *p* < 0.05 and ** *p* < 0.01 vs. the MC group.

In the present study, the histomorphology of the spleen and thymus were examined with an optical microscope. The spleens (Figure 1A) and thymuses (Figure 1B) of normal control mice displayed massive closely arranged and deeply stained splenocytes and thymocytes with an obvious nucleus. In the model (MC) group, we observed a decrease in the number of splenocytes and thymocytes. In addition, there were necrotic areas with no cell structures in the HE stained histopathological images. While in the medium (Be-M) and high (Be-H) bergenin groups, the spleen and thymus cells were arranged compactly in good order with clear nuclei and less intercellular space, which were similar to those in the normal group. The results indicate that bergenin significantly prevented the damage to the spleen and thymus in the mice induced by Cy.

### 2.2. Body Weights and Immune Organ Index 

As shown in Figure 1C,D, there was a remarkable decrease in body weights in the model group after challenging. While administrated with bergenin, the weights of the mice recovered significantly. As shown in Figure 1E, compared with the normal group, the spleen and thymus indices significantly decreased (*p* < 0.01) in the Cy-treated mice. After administrated with bergenin, there was the significant recovery of spleen and thymus indices compared with the model group (*p* < 0.05 or *p* < 0.01). The degree of recovery in the spleen was more pronounced than that in the thymus. 

### 2.3. Effect of Bergenin on Cytokine Levels in Serum of Cy-Treated Mice

IFN-γ and IL-2 are generally called as Th1-type cytokines while IL-4 and IL-10 are generally defined as Th2-type cytokines [26]. The balance of Th1/Th2 was analyzed by the following formula:

(1) Th1/Th2=(IFN-γ+IL-2)/(IL-4+IL-10)

**Figure 2:**
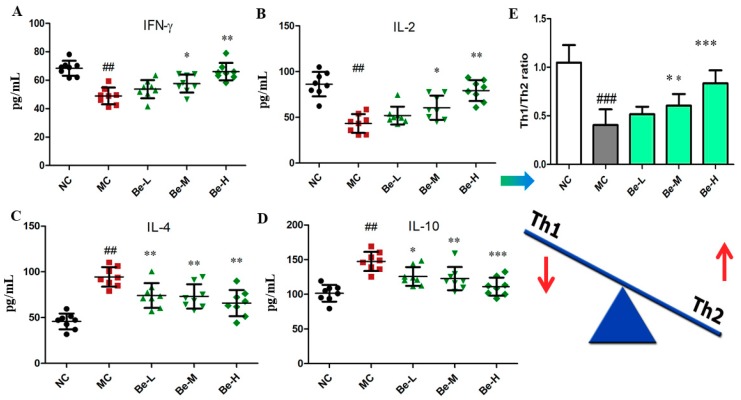
Effect of bergenin on cytokines in the Cy-treated mice serum. (**A**) Serum IFN-γ levels; (**B**) Serum IL-2 levels; (**C**) Serum IL-4 levels; (**D**) Serum IL-10 levels; (**E**) Th1 and Th2 cytokine ratio. NC: normal control group, dosed with saline; MC: model control group, challenged with cyclophosphamide; Be-L: challenged with Cy and treated with 5 mg/kg/day bergenin; Be-M: challenged with Cy and treated with 10 mg/kg/day bergenin; Be-H: challenged with Cy and treated with 20 mg/kg/day bergenin. Data are expressed as mean ± SD (*n* = 8). ^##^ *p* < 0.01 and ^###^ *p* < 0.001 vs. the NC group, * *p* < 0.05, ** *p* < 0.01 and *** *p* < 0.001 vs. the MC group.

We found that cyclophosphamide injection caused a significant reduction in IFN-γ (*p* < 0.01) and IL-2 (*p* < 0.01) in the sera of the model group while the expressions of IL-4 (*p* < 0.01) and IL-10 (*p* < 0.01) in the sera of the model group were obviously higher than those of the normal group. As shown in Figure 2, bergenin significantly prevented the Cy-induced reduction in the Th1-type cytokines (IFN-γ and IL-2) in the sera. In addition, bergenin remarkably reversed the Cy-induced increase of the Th2-type cytokines (IL-10 and IL-4) in the sera. Figure 2E shows that Th1/Th2 cytokine ratios in the model group were significantly lower than that in the normal group (*p* < 0.001). While administrated with bergenin at the doses of 10 and 20 mg/kg (Be-M, Be-H) significantly restored the Th1/Th2 balance (*p* < 0.05 or *p* < 0.001). 

### 2.4. Effect of Bergenin on Immunoglobulin Levels in Serum

**Figure 3:**
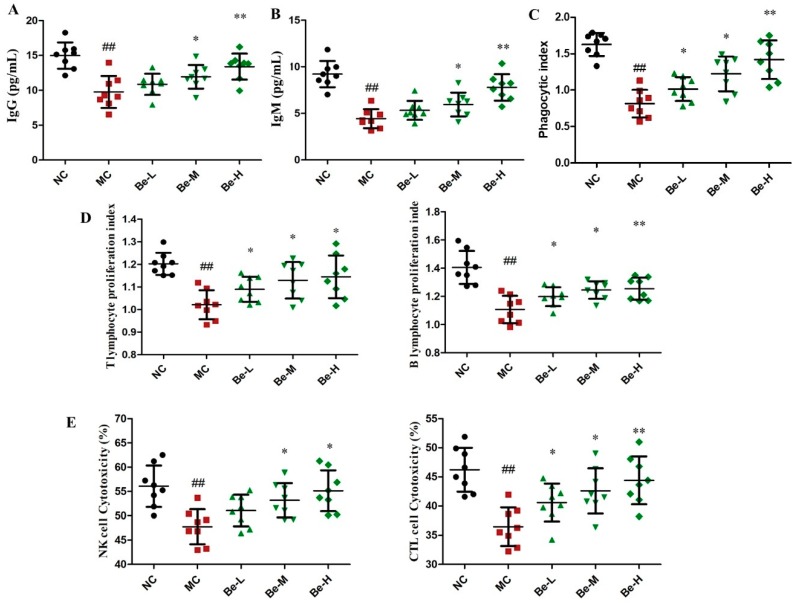
Effect of bergenin on immunoglobulin levels and immune cell activity in the Cy-treated mice. (**A**) Serum IgG levels; (**B**) Serum IgM levels; (**C**) Peritoneal macrophage phagocytosis; (**D**) Splenocyte proliferation induced by Con A and LPS; (**E**) Splenic NK and CTL cytotoxic activities. NC: normal control group, dosed with saline; MC: model control group, challenged with cyclophosphamide; Be-L: challenged with Cy and treated with 5 mg/kg/day bergenin; Be-M: challenged with Cy and treated with 10 mg/kg/day bergenin; Be-H: challenged with Cy and treated with 20 mg/kg/day bergenin. Data are expressed as mean ± SD (*n* = 8). ^##^ *p* < 0.01 vs. the NC group, * *p* < 0.05 and ** *p* < 0.01 vs. the MC group.

As shown in Figure 3A,B, we found that the productions of IgG (*p* < 0.01) and IgM (*p* < 0.01) in the sera were noticeably suppressed in the model control compared with the normal control. Bergenin at the concentrations of 10 and 20 mg/kg were found to significantly prevent the decline in the levels of IgG and IgM. 

### 2.5. Effect of Bergein on Phagocytic Activity of Macrophages in Cy-Treated Mice

As shown in Figure 3C, Cy caused an obvious inhibition of the phagocytosis of macrophage from 1.66 to 0.83 (*p* < 0.01). The absolute rate reduction of macrophage phagocytosis was 48.2% in the Cy-treated mice. However, bergenin (Be-L, Be-M, Be-H) dramatically reversed the impaired phagocytosis capacity of peritoneal macrophages in a dose dependent manner (*p* < 0.05 or *p* < 0.01).

### 2.6. Effects of Bergenin on Splenocyte Proliferation in Cy-Treated Mice

As shown in Figure 3D, the proliferation responses of spleen lymphocytes to T cell-Con A and B cell-LPS stimuli were slightly but significantly inhibited in the Cy-treated mice compared with the normal group. Bergenin at three doses (Be-L, Be-M, Be-H) moderately elevated responses of both T and B lymphocytes in the Cy-treated mice *(p <* 0.05 or *p <* 0.01).

### 2.7. Effects of Bergenin on Splenic NK and CTL Cytotoxicities in Cy-Treated Mice

NK and CTL activities were slightly but significantly inhibited in the Cy-treated mice compared to the normal group (Figure 3E). Treatment with bergenin significantly increased the NK and CTL cytotoxic activities at three doses (Be-L, Be-M, Be-H) in the Cy-treated mice (*p* < 0.05 or *p* < 0.01). Especially at the high dose of bergenin (Be-H), the activities of NK and CTL were increased to a value almost equal to the normal control group.

### 2.8. Effects of Bergenin on Splenic T Lymphocyte Subsets 

**Figure 4:**
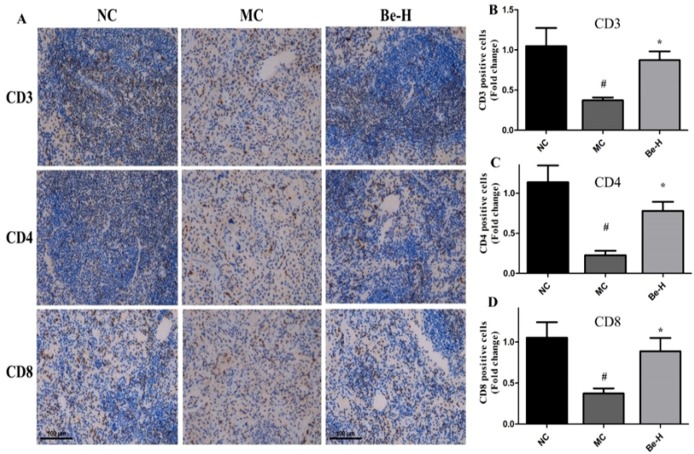

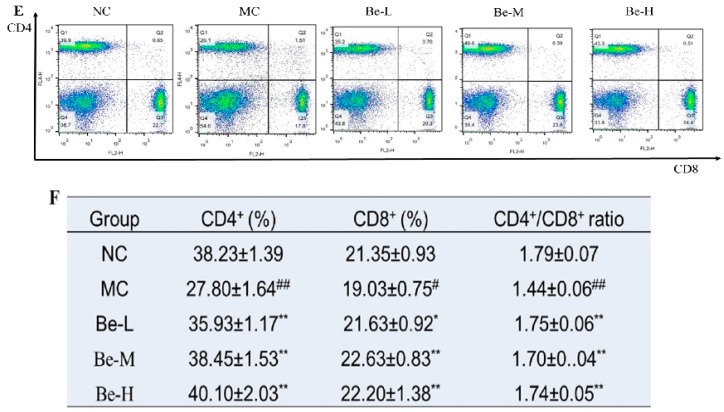
Effects of bergenin on splenic T lymphocyte subsets in the Cy-treated mice. (**A**) CD3^+^, CD4^+^ and CD8^+^ T lymphocyte expression in spleen tissues. (**B**) Bar graphs represent mean ± SEM of the numbers of CD3^+^-positive cells from five microscopic fields in each group. (**C**) Bar graphs represent mean ± SD of the numbers of CD4^+^-positive cells from five microscopic fields in each group. (**D**) Bar graphs represent mean ± SEM of the numbers of CD8^+^-positive cells from five microscopic fields in each group. (**E**) Splenic T lymphocyte subsets detected by flow cytometry. (**F**) Splenic T lymphocyte subpopulation analyzed by flow cytometry. NC: normal control group, dosed with saline; MC: model control group, challenged with cyclophosphamide; Be-L: challenged with Cy and treated with 5 mg/kg/day bergenin; Be-M: challenged with Cy and treated with 10 mg/kg/day bergenin; Be-H: challenged with Cy and treated with 20 mg/kg/day bergenin. Values are shown as mean ± SD (*n* = 8). ^#^ *p* < 0.05, ^##^ *p* < 0.01 vs. the NC group, * *p* < 0.05 and ** *p* < 0.01 vs. the MC group.

On the basis of the effect of bergenin on lymphocyte cell activities, splenic T lymphocyte subsets were detected by immunohistochemical analysis and flow cytometry. As seen in Figure 4A, positive cells were observed as dark brown-stained regions. The immunohistochemical assay showed that the counts of CD3^+^, CD4^+^ and CD8^+^ T lymphocytes were distinctly depleted in the model group (Figure 4B–D). Bergenin at the high dose reversed all of them. As shown in Figure 4E,F, the percentages of CD4^+^ and CD8^+^ T lymphocytes in the model group were significantly lower than those in the normal group. Cy had a stronger inhibitory effect on CD4^+^ T lymphocytes and dramatically reduced the ratio of CD4^+^/CD8^+^ (*p* < 0.01). The results show that a significant augment in CD4^+^ and CD8^+^ T lymphocyte subsets in the mice treated with bergenin at three doses (Be-L, Be-M, Be-H) compared to the model group. 

### 2.9. Effect of Bergenin on Redox Imbalance in Cy-Treated Mice

**Figure 5:**
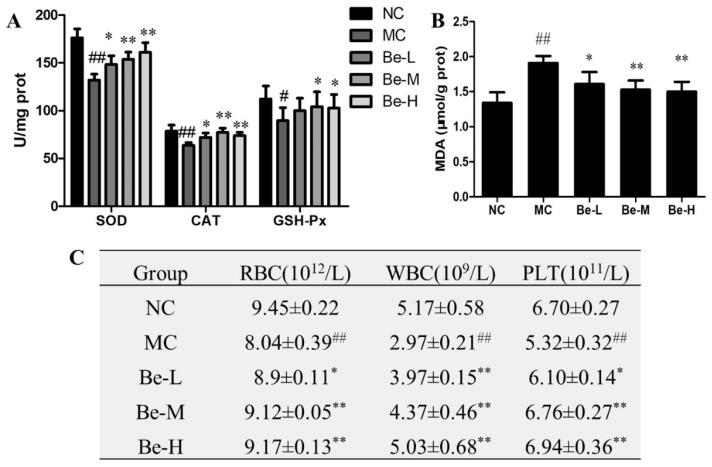
Antioxidative activity in spleens and blood cells counts changes after cyclophosphamide injection. (**A**) Level of SOD, CAT and GSH-Px; (**B**) Level of MDA; (**C**) Blood cells counts. NC: normal control group, dosed with saline; MC: model control group, challenged with cyclophosphamide; Be-L: challenged with Cy and treated with 5 mg/kg/day bergenin; Be-M: challenged with Cy and treated with 10 mg/kg/day bergenin; Be-H: challenged with Cy and treated with 20 mg/kg/day bergenin. Values are expressed as mean ± SD, *n* = 8 mice per group. ^#^ *p* < 0.05, ^##^ *p* < 0.01 vs. the NC group, * *p* < 0.05 and ** *p* < 0.01 vs. the MC group.

To further explore the protection mechanisms of bergenin in the Cy-treated mice, we determined the activities of antioxidant enzyme including SOD, GSH-Px and CAT as well as the level of MDA in the mouse spleens of all the groups studied. The activities of SOD (*p* < 0.05), CAT (*p* < 0.05) and GSH-Px (*p* < 0.01) of the model group were distinctly lower than those of the normal group (Figure 5A) whereas bergenin at the doses of 10 and 20 mg/kg reversed all of them. Moreover, bergenin at three doses effectively weakened the elevation of MDA induced by Cy in varying degrees (Figure 5B). 

### 2.10. Effect of Bergenin on RBC, WBC and Platelets Count in Cy-Treated Mice

To evaluate the hemopoietic function, we examined the effects of bergenin on RBC, WBC and platelets counts in the Cy-treated mice. As shown in Figure 5C, compared to the normal group, the counts of RBC, WBC and platelets from peripheral blood were significantly decreased by Cy (*p* < 0.01). The decline in leukocyte number was the most serious from 5.17 ± 0.58 to 2.97 ± 0.21 (10^9^/L). The reduced counts of RBC, WBC and platelets were elevated remarkably by bergenin. Treatments with bergenin at the high dose (20 mg/kg) restored WBC and RBC counts nearly to the normal levels. 

## 3. Discussion

Immunosuppression is a state of temporary or permanent immunity dysfunction and can make organisms more sensitive to pathogens due to the damage of the immune system [27]. It is an area of active interest for discovering immunomodulatory agents from medicinal plant origins for immunosuppressive diseases treatment. A large number of Traditional Chinese medicines have been shown to possess immunomodulating activities [12,13]. *Ardisia japonica* has been employed clinically with the combination of other Chinese herbal medicines to treat patients with tuberculosis by improving host immunity [28]. Bergenin is the main constituent of *Ardisia japonica*. Thus, this study was designed to assess the effect of bergenin on the immune system in the cyclophosphamide (Cy)-induced mice. 

It is well known that Cy is a common cytotoxic drug used widely in the tumor treatment [25,29]. Its cytotoxic effects are the result of chemically reactive metabolites that alkylate DNAs and proteins by producing cross-linking [25]. It has been reported that Cy can cause atrophy and weight loss of the immune organs and an imbalance of various leukocytes in the peripheral blood of mice, eventually inhibiting immune function [30,31]. Therefore, morphology of the spleen and thymus, immune organ indexes and blood cell counts were examined in this study. Our data demonstrate that bergenin prevented the decline in thymus and spleen indexes induced by Cy. The thymus and spleen are important immune organs, which are the major players in maintaining immune homeostasis [32]. The results of HE staining in the spleen and thymus (Figure 1A,B) show that bergenin could prevent the damage to spleen and thymus induced by Cy, indicating that bergenin is able to counteract the effect of immunosuppression on immune organs. In addition, compared with the Cy-treated mice, the total RBC, WBC and platelet counts were increased upon the administration of bergenin, thereby providing supportive evidences for its anti-anemic, anti-leukopenic, and anti-thrombocytopenic activities.

Cy can kill immune cells as well as interfere with the proliferation and differentiation of T and B cells. It has been reported that Cy can significantly inhibit the humoral and cellular immune responses [33,34].The consequence of the adverse effects of Cy on the monocytes and macrophages that regulate the functions of lymphocytes leads to the reduced proliferative capacities of T and B lymphocyte populations in rats [35]. NK cells can stimulate the production of cytokines and CTL cell kill tumor cells by direct cytotoxicity [36]. Cy has been shown to suppress NK and CTL cell activities [28]. Compared with the model group, the phagocytosis of peritoneal macrophages, proliferation of T and B splenocytes and the activities of NK and CTL cells in the bergenin-treated groups have been improved and the immune activity of mice with immunosuppression have been increased. CD4^+^ and CD8^+^ are T helper (Th) and T cytotoxic (Tc) lymphocytes, which are the most important immune cells in the regulating component of immune systems via releasing pro-inflammatory cytokines or direct cytotoxic effects [37,38]. Previous studies have shown that the ratio of CD4^+^ and CD8^+^ T lymphocytes in immunosuppressed mice was lower than normal mice [37]. Our results show that bergenin possessed the capability of preventing the Cy-induced decrease in the proportions of CD3^+^, CD4^+^ and CD8^+^ T lymphocyte subsets. Based on the above results, bergenin had multiple positive effects to enhance cellular immunity in the Cy-treated mice. IgG and IgM are the major immunoglobulins that can enhance the humoral immunity to defense against all kinds of external pathogens [28]. As shown in this work, the data indicate that bergenin could enhance humoral immunity by promoting the production of IgG and IgM.

In a healthy immune system, there is a dynamic balance between Th1 and Th2 cell activity to keep normal cellular and humoral immune functions [26,39]. Cytokines play a pivotal role in immune responses. Cy suppresses the secretion of Th1-type cytokines and increases the secretion of Th2-type cytokines [10]. IFN-Υ is one of the major cytokines produced by Th1 cells, such as activated CD4^+^ T cells, CD8^+^ T cells and NK cells, and contributes to cell-mediated inflammatory immune responses [40]. Our results show that bergenin was able to significantly reverse the increase of serum Th2-type cytokines (IL-4 and IL-10), and decline of Th1-type cytokines (IL-2 and IFN-γ), indicating that bergenin possessed the capability of modulating the Th1/Th2 balance. Therefore, the reasons why the cytokines in the bergenin group were restored to the levels of the control group may be related to the proliferation of T cells. 

The oxidative DNA damage in cells is the main side effect of chemotherapy drugs including cyclophosphamide [6]. The modulation of oxidative stress and inflammatory cytokine production through stimulation of immunity may help in maintaining a disease-free state [35]. It has been reported that the oxidative stress mediates disruption of the redox balance after Cy exposure [41]. It is well-known that the activities of antioxidative enzymes such as SOD, GSH-Px, CAT and oxidative enzymes such as MDA are considered as the common indexes of tissue antioxidant status [42]. It has been reported that bergenin either acts as a free radical scavenger or a redox regulatory agent. In addition, free radical scavenging activity and antioxidant potential of bergenin on c-radiation induced liposomal lipid peroxidation, protein carbonylation and DNA damage are also investigated [43]. Our results show that the administration with bergenin reversed the decline in the activities of SOD, CAT and GSH-Px as well as elevation of MDA in the Cy-treated mouse spleens. Therefore, our findings indicate that bergenin effectively protected the oxidative stress injury induced by Cy by rising antioxidative enzymes and reducing oxidative enzymes.

A lot of Traditional Chinese Medicines such as Astragalus polysaccharides have been proved to modulate immunity through the TLR4 signaling pathway and eventually to participate in the regulation of nuclear factor kappa B (NF-κB) activation [44]. While the present study demonstrated that bergenin plays an anti-inflammatory role via the modulation of MAPK and NF-κB signaling pathways in a mouse model of LPS-induced mastitis [45]. In addition, the previous study shows that bergenin induces Th1 immune responses and potently inhibits bacillary growth in a murine model of Mycobacterium tuberculosis infection by activating the MAP kinase and ERK pathways [46]. Therefore, we speculate that the mechanism of the immunomodulatory action bergenin may be though the NF-κB and MAPK pathways. In summary, we have provided the evidences that bergenin could effectively improve the immune functions including humoral and cellular immunity and raised the antioxidative activities in the Cy-treated mice. Our results suggest that bergenin might be a potent natural immunomodulatory agent, which could be evaluated for a clinical application in patients with immunosuppression induced by the chemotherapy.

## 4. Materials and Methods 

### 4.1. Materials and Reagents

**Figure 6:**
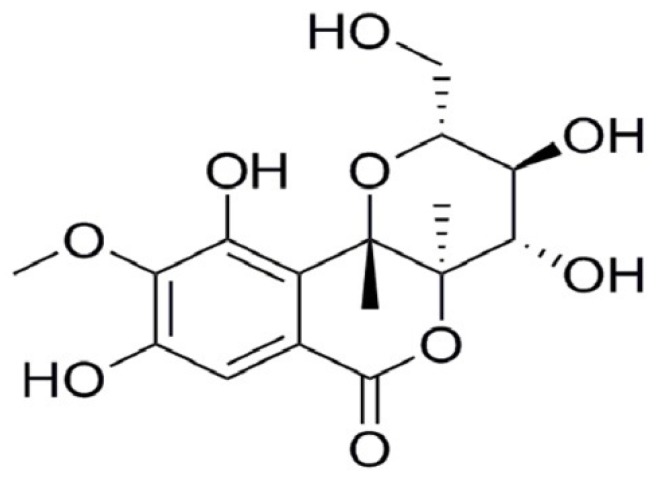
The chemical structure of bergenin.

Bergenin (purity of 95%) was obtained from Meilun Biological Products Co. Ltd. (Dalian, China). The chemical structure of bergenin is shown in Figure 6. The cell culture products were from Gibco BRL (Grand Island, NY, USA). Injectable cyclophosphamide was obtained from Jiangsu Hengrui Medicine Co. (Lianyungang, Jiangsu, China). A HE staining kit was purchased from Beyotime (Jiangsu, China). Concanavalin A (Con A), lipopolysaccharide (LPS) and neutral red (N7005) were provided by Sigma-Aldrich (St. Louis, MO, USA). The Ig kit and cytokines ELISA kits were purchased from Lianke Biotechnology Co. Ltd. (Hangzhou, China). SOD, CAT, GSH-Px and MDA detection kits were provided by Nanjing Jiancheng Bio-engineering Institute (Nanjing, China). 2-(2-Methoxy-4-nitrophenyl)-3-(4-nitrophenyl)-5-(2,4-disulfophenyl)-2H-tetrazolium, mono- sodium salt] (WST-8) was purchased from Amresco Co. (Solon, OH, USA) Anti-CD4^+^ (APC) and anti-CD8^+^ (PE) antibodies were provided by Bio Legend, Inc. (San Diego, CA, USA). 

### 4.2. Animals and Experimental Design 

Male Balb/c mice (6–8 weeks old, 18–20 g) were obtained from Huafukang Biological Products Co. Ltd. (Beijing, China). These mice were housed in a rodent facility at 25 °C with a 12-h light-dark cycle for acclimatization. All procedures involving animal care were approved by the Ethics Committee of Shandong University (No. 2016020, Jinan, China). After being adapt to environment for one week, these mice were randomly divided into 5 groups consisting of 8 mice each. Mice were treated as Table 1**.** One group of healthy mice were used as a normal control group (NC) treated once daily with the physiological saline solution by intraperitoneal injection (i.p.) for 10 days. From days 1 to 3 and 9, the other groups were given 80 mg/kg/day cyclophosphamide (i.p.). From day 4 to 10, the Cy-treated mice were administered as the following (i.p.): the physiological saline solution (MC group), 5, 10 and 20 mg/kg/day bergenin (Be-L, Be-M, Be-H group). Bergenin dissolved in the physiological saline. The mouse body weights were recorded before the daily administration and during the study period. At the 24-h time point after the last dose, the animals were weighed and then killed.

### 4.3. HE Staining of Spleen and Thymus Tissues

After isolated from mice, spleen and thymus tissues were fixed with 4% paraformaldehyde, embedded in paraffin and cut into 4-μm sections. Then these sections were deparaffinized and stained with hematoxylin and eosin. Finally, the sections were dehydrated and mounted for imaging. 

### 4.4. Effect of Bergenin on Mouse Spleen and Thymus Indices

At the 24-h time point after the last intraperitoneal injection, the mice were weighed and then sacrificed via decapitation and then thymus and spleen of each mouse were collected and immediately weighed to calculate the immune organ indices according to the following formula:

(2) $$\mathrm{Index}\;\left(\mathrm{mg}/g\right)=\frac{\mathrm{weight}\;\mathrm{of}\;\mathrm{spleen}\;\mathrm{or}\;\mathrm{thymus}}{\mathrm{body}\;\mathrm{weight}}$$

### 4.5. Determination of Immunoglobulin and Cytokines in Serum by ELISA

Serum samples were prepared by centrifuging the whole blood at 3500 rpm at 4 °C for 10 min. The levels of immunoglobulin (IgG and IgM) and cytokines for IFN-γ, IL-2, IL-4, and IL-10 in the serum were measured according to the ELISA kit instructions.

### 4.6. Phagocytosis of Peritoneal Macrophages

Macrophages were prepared from Balb/c mice in order to evaluate the phagocytosis of peritoneal macrophages using the neutral red uptake as previously described [47,48]. Peritoneal macrophages of Balb/c mice were aseptically harvested by the peritoneal lavage with 5 mL PBS. They were seeded in 96-well plate with a complete RPMI-1640 medium at a density of 5 × 10^5^/well and cultured at 37 °C in 5% CO_2_ for 24 h. The cells were washed with PBS for three times and 100 µL of the neutral red solution (0.075%) was added and incubated for another 1 h. Then the cells were washed with PBS for three times to remove excess dye and incubated with a cell lysis buffer (1% acetic acid: ethanol = 1:1) (100 μL/well). The absorbance was detected at 540 nm by a microplate reader. 

### 4.7. Measurement of Lymphocyte Proliferation

The splenic lymphocytes were separated from Balb/c mouse spleens in order to analyze the lymphocyte proliferation using a CCK8 assay as described previously [28]. Briefly, splenocytes were obtained by milling the spleen and filtering by a sieve mesh and then added to the lysis buffer, held for 10 min to take out red blood cells and washed with the cold PBS for three times. After centrifuging at 200× *g* for 5 min, single splenocyte was resuspended in the RPMI-1640 and adjusted to the concentration of 2 × 10^6^/mL. 100 μL of cell suspension was added to the wells of 96-well plates with or without 5 μg/mL of Con A or 10 μg/mL LPS incubated at 37 °C in 5% CO_2_ for 70 h. Then 10 μL of WST-8 (5 mg/mL) was added to each well incubated for another 2 h and then the absorbance was detected at 450 nm by a microplate reader. 

### 4.8. Splenic NK and CTL Cell Cytotoxicity Activity Assays

Splenic lymphocytes obtained from the spleen were used as the effector cells for splenic NK and CTL cell activity assays as described above [49]. YAC-1 and HL-60 cells were used as the target cells for NK and CTL cells, respectively. Briefly, the effector cells (5 × 10^5^/well) and target cells (1 × 10^4^/well) were mixed at a ratio of 50:1, seeded into 96-well plates and incubated for 22 h at 37 °C in 5% CO_2_. Then 10 μL of WST-8 (5 mg/mL) was added to each well, incubated for another 2 h and then the absorbance was detected at 450 nm by the microplate reader. The cytotoxic activity was calculated according to the following formula:

(3) $$\mathrm{cytotoxicity}\;\left(\%\right)=\frac{T_{\mathrm{OD}}-\left(S_{\mathrm{OD}}-E_{\mathrm{OD}}\right)}{T_{\mathrm{OD}}}\times 100\%$$

where T_OD_ is an optical density value of the target cell control, S_OD_ is an optical density value of test samples, and E_OD_ is an optical density value of the effector cell control.

### 4.9. Immunohistochemical Analysis of CD3, CD4 and CD8 Expressions in Spleen Tissues

Freshly dissected tissues were fixed and embedded in paraffin. After being cut into 4-µm slices, the sections were incubated with anti-mouse CD3, CD4 or CD8 monoclonal antibodies (Biolegend, San Diego, CA, USA) overnight at 4 °C. HRP-tagged goat anti-rat IgG (ZSGB-BO Co., Ltd., Beijing, China) was added as a secondary antibody for 30 min at room temperature. Then, the sections were treated with 3,3-diaminobenzidine (DAB) and stained with hematoxylin. Finally, the sections were dehydrated, cleared, and mounted with neutral gum for imaging.

### 4.10. Flow Cytometry Analysis of Splenic T Lymphocyte Subpopulations

The spleen lymphocytes were prepared and adjusted to 1 × 10^7^/mL and incubated with anti-CD4^+^ (APC) 0.4 µL and anti-CD8^+^ (PE) 0.3 µL for 30 min at 4 °C under dark. The spleen lymphocytes were washed for three times with PBS. Then the ratios of CD4^+^ and CD8^+^ T lymphocyte subpopulations were analyzed by flow cytometry (Beckman Coulter FC500, Miami, FL, USA).

### 4.11. Measurements of Antioxidant Enzyme Activities and the MDA Level

Spleen tissues (100 mg) were prepared with the phosphate buffer saline (50 mM, pH 7.4) using a Teflon homogenizer. The homogenate was then centrifuged at 12,000 rpm for 10 min at 4 °C and the supernatants were then used for the measurements of activities of SOD, CAT, GSH-Px and MDA. All biochemical parameters were measured with assay kits (Nanjing Jiancheng Bioengineering Institute, Nanjing, China) according to the manufacturer’s protocols. 

### 4.12. Peripheral White Blood Cell, Red Blood Cell and Platelets Counts

Blood was collected by a retro-orbital bleeding method into heparin tubes on the day of sacrifice. The red blood cell (RBC), white blood cell (WBC) and platelet differential counts were detected via a LH755 Hematology Analyzer (Coulter, Miami, FL, USA).

### 4.13. Statistical Analysis

Data are showed as mean ± SD. The statistical significance of the differences between various groups were determined by either a Student t-test or an ANOVA analysis for multiple comparisons by Prism version 5.0 (GraphPad Software, Inc., La Jolla, CA, USA. Values of *p* < 0.05 were considered statistically significant.

## Figures and Tables

**Table 1:** Mouse treatment schedule. NC: normal control group, dosed with saline; MC: model control group, challenged with cyclophosphamide; Be-L: challenged with Cy and treated with 5 mg/kg/day bergenin; Be-M: challenged with Cy and treated with 10 mg/kg/day bergenin; Be-H: challenged with Cy and treated with 20 mg/kg/day bergenin.

Group	Treatment (i.p.)	
	Days 1–3 and 9	Days 4–10
NC	Normal saline	Normal saline
MC	Cy (80 mg/kg/day)	Normal saline
Be-L	Cy (80 mg/kg/day)	Bergenin (5 mg/kg/day)
Be-M	Cy (80 mg/kg/day)	Bergenin (10 mg/kg/day)
Be-H	Cy (80 mg/kg/day)	Bergenin (20 mg/kg/day)
